# Amphidromy Links a Newly Documented Fish Community of Continental Australian Streams, to Oceanic Islands of the West Pacific

**DOI:** 10.1371/journal.pone.0026685

**Published:** 2011-10-21

**Authors:** Paul A. Thuesen, Brendan C. Ebner, Helen Larson, Philippe Keith, Rebecca M. Silcock, Jason Prince, David J. Russell

**Affiliations:** 1 School of Marine and Tropical Biology, James Cook University, Cairns, Queensland, Australia; 2 Department of Employment Economic Development and Innovation, Cairns, Queensland, Australia; 3 Australian Rivers Institute, Griffith University, Nathan, Queensland, Australia; 4 Museum and Art Gallery of the Northern Territory, Darwin, Northern Territory, Australia; 5 Muse'um National d'Histoire Naturelle, Laboratoire d'ichtyologie, Paris, France; 6 The Patch, Melbourne, Victoria, Australia; Institute of Marine Research, Norway

## Abstract

**Background:**

Indo-Pacific high island streams experience extreme hydrological variation, and are characterised by freshwater fish species with an amphidromous life history. Amphidromy is a likely adaptation for colonisation of island streams following stochastic events that lead to local extirpation. In the Wet Tropics of north-eastern Australia, steep coastal mountain streams share similar physical characteristics to island systems. These streams are poorly surveyed, but may provide suitable habitat for amphidromous species. However, due to their ephemeral nature, common non-diadromous freshwater species of continental Australia are unlikely to persist. Consequently, we hypothesise that coastal Wet Tropics streams are faunally more similar, to distant Pacific island communities, than to nearby faunas of large continental rivers.

**Methods/Principal Findings:**

Surveys of coastal Wet Tropics streams recorded 26 species, 10 of which are first records for Australia, with three species undescribed. This fish community is unique in an Australian context in that it contains mostly amphidromous species, including sicydiine gobies of the genera *Sicyopterus, Sicyopus, Smilosicyopus* and *Stiphodon*. Species presence/absence data of coastal Wet Tropics streams were compared to both Wet Tropics river networks and Pacific island faunas. ANOSIM indicated the fish fauna of north-eastern Australian coastal streams were more similar to distant Pacific islands (R = 0.76), than to nearby continental rivers (R = 0.98).

**Main Conclusions/Significance:**

Coastal Wet Tropics streams are faunally more similar to distant Pacific islands (79% of species shared), than to nearby continental fauna due to two factors. First, coastal Wet Tropics streams lack many non-diadromous freshwater fish which are common in nearby large rivers. Second, many amphidromous species found in coastal Wet Tropics streams and Indo-Pacific islands remain absent from large rivers of the Wet Tropics. The evolutionary and conservation significance of this newly discovered Australian fauna requires clarification in the context of the wider amphidromous fish community of the Pacific.

## Introduction

Freshwater fish faunas of the Indo-Pacific islands differ to their continental counterparts. Island streams are generally lower in species richness, and are characterised by a high proportion of amphidromous species [1], [2], [3]. Species with an amphidromous life history (whereby hatched embryos are swept out to sea to develop as pelagic larvae before recruiting back to streams as post-larvae) remain mostly absent from large continental rivers. Newly hatched amphidromous larvae consume oceanic plankton and are physiologically adapted for development in seawater, as a consequence, larval survival depends on rapid transport to the sea [1], [4], [5], [6]. Only steep, short and swiftly flowing coastal streams that drain directly into the ocean, such as those on Indo-Pacific islands, provide suitable habitat for many amphidromous species. Slower moving rivers with large lowland and estuarine components represent impassable barriers to migration, resulting in larval starvation [7]. One group of amphidromous fishes that commonly inhabit steep island streams are the gobies of the sub-family Sicydiinae (family Gobiidae). Sicydiines contribute significantly to the diversity of Indo-Pacific and Caribbean streams, and often comprise the highest levels of endemism [1], [8]. This group of fishes also possess the ability to climb waterfalls, sometimes hundreds of meters high [9].

Amphidromy is an important adaptation for the re-colonisation of island habitats which are often ephemeral in nature [10], [11]. Small island streams are subject to extreme climatic and hydrological variation, and are likely to dewater from climatic fluctuations [12], [13], [14]. In addition, island faunas may be subjected to local extirpation over geologic timescales [15], [16], due to the formation and disappearance of land at subduction zones and hot spots [17]. As a consequence, the pelagic larval phase of amphidromous species is a crucial dispersal and colonisation mechanism between ephemeral island streams.

In the Wet Tropics region of north-eastern Australia, there exist large rivers typical of continental landmasses, and small coastal streams draining high mountain ranges. These latter systems are similar in their geomorphology and hydrology to oceanic island streams in that they are small coastal hydrological units (stream order ≤3), of steep gradient, discharging directly into the sea without passing through an extensive estuary/lowland component. To date, freshwater fish surveys of the Australian Wet Tropics have mostly targeted larger river systems (stream order ≥5) and their feeder stream networks [18], [19], [20], or small adventitious coastal streams (feeder streams at least three orders smaller in magnitude than mainstreams) which drain lowlands [19]. The species richness of these large rivers is considered relatively high in comparison to drier catchments of Australia [21] and the freshwater fish community is characterised by mostly catadromous and non-diadromous species (i.e. species which are obligate to freshwaters for their entire life history). In contrast, coastal Wet Tropics streams remain relatively poorly surveyed. Pusey and Kennard [22] concluded that the these streams (i.e. at Cape Tribulation), were faunally least similar to other rivers and their stream networks of the Wet Tropics region, with the fish community composed primarily of diadromous species and marine vagrants. Recent preliminary surveys of coastal Wet Tropics streams have detected the presence of sicydiine gobies and noted a lack of non-diadromous species common to the larger rivers of the area [23], [24], [25].

In this study, we undertook the first comprehensive fish community survey of five coastal Australian Wet Tropics streams. The species compositions of these streams were compared with published fish community datasets from the larger rivers of the Wet Tropics and the broader West Pacific island faunas. We hypothesised that coastal Wet Tropics streams are faunally more similar to distant Pacific Island communities than to nearby faunas of large Wet Tropics rivers. Specifically, coastal Wet Tropics streams are likely to possess a high proportion of amphidromous species such as sicydiine gobies, while lacking many of the more common non-diadromous freshwater species which inhabit the larger rivers of the region. Such a pattern could be attributed to 1) the strict habitat requirements of some amphidromous larvae such as sicydiines (i.e. needing immediate access to the sea to avoid larval starvation), which exclude their persistence in rivers with large estuarine systems, and 2) the likely ephemeral nature of small Wet Tropics streams, which will exclude many non-diadromous freshwater species commonly found in the larger rivers of the region.

## Materials and Methods

### Ethics Statement

Permission to undertake field work at the study sites and collect specimens was obtained under General Fisheries Permit 89212 and Environment Protection Agency Permit WITK06337909. Specimens were obtained under Griffith Animal Ethics Committee approval ENV114/09/AEC and ENV10/09/AEC.

### Study Sites

**Figure 1:**
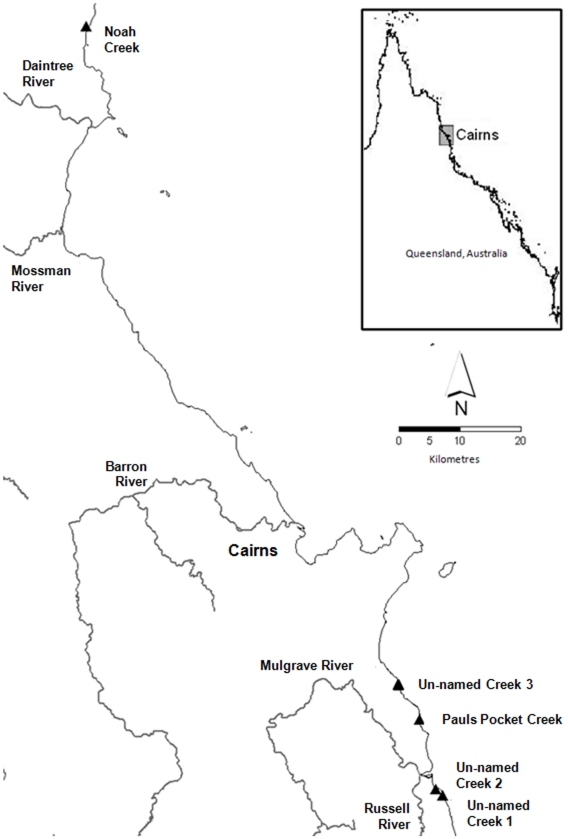
Map of study sites in north-eastern Australia. Location of five study sites in the Wet Tropics of north-eastern Australia. Solid triangles denote where freshwater fish surveys were conducted.

Five non-adventitious coastal streams in the Wet Tropics of north-eastern Australia were chosen for survey. For the sake of brevity, these streams will be referred to as coastal Wet Tropics streams in this study. Streams were selected for survey based on the following criteria: a small stream order (≤3), a steep gradient, the absence of an extensive estuary, an intact rainforest riparian zone and relatively perennial flows. Streams were selected to encompass the range of catchment sizes that drain these steep coastal ranges (Table 1). Noah Creek, located north of the regional city of Cairns, was the largest catchment investigated (29.8 km^2^) (Fig. 1). This system drains the northern flank of Thornton Range and has a small lowland component to its catchment which includes a small estuarine zone. Pauls Pocket Creek and Un-named Creek 1 drain the Malbon Thompson range to the south of Cairns. These systems have no significant lowland habitat, however, Pauls Pocket Creek flows into a very small coastal lagoon which remains closed to the ocean during periods of minimal flow. Un-named Creek 2 and Un-named Creek 3 flow off the eastern ridge of the Graham Range to the south of Cairns. Both of these catchments are very small and steep (1.2 km^2^ and 0.4 km^2^ respectively) with no lowland/estuarine component. All study streams are in a relatively pristine state with no artificial barriers to flow, although water is abstracted from Un-named Creek 2 for the small coastal community of Russell Heads.

**Table 1:** Physical characteristics of five coastal north-east Australian Wet Tropics streams.

Site	Length: source to mouth (km)	Estuary length (km)	Catchment area (km^2^)	Average gradient (m/km)	Source elevation (m)
Noah Creek	12.8	2.5	29.8	133.4	1370
Pauls Pocket Creek	4.9	0.2	5.8	166.0	780
Un-named Creek 1	3.1	0.0	3.8	224.2	695
Un-named Creek 2	2.0	0.0	1.2	303.5	605
Un-named Creek 3	0.9	0.0	0.4	444.0	400

All three coastal ranges investigated are of granitic composition and were formed during the Late Tertiary due to coastal scarp retreat combined with differential weathering following tilting of the continental margin [26]. Precise climate data from each site were unavailable. However, mean precipitation for nearby Cape Tribulation (Thornton Range) and Bellenden Ker (Malbon Thompson and Graham Range) is high (∼3990 mm.yr^−1^) (Commonwealth of Australia, Bureau of Meteorology) due to the oblique orientation of the three mountain ranges relative to the prevailing south-easterly trade winds. The vegetation at each site is classified by Tracey and Webb [27] as type 2a mesophyll vine forest (i.e. structurally complex rainforest on very wet & wet lowlands and foothills (<400 m a.s.l) on granites and metamorphics).

### Survey technique

In the Wet Tropics of north-eastern Australia, previous freshwater fish studies have mostly used electro-fishing as a survey technique [18], [19]. Electro-fishing effectiveness is highly variable across species and is usually more effective on larger, migratory fishes [28]. In contrast, its efficacy is considered poor for the collection of gobiids [25], [29], as most do not possess a swim bladder and are negatively buoyant. Therefore, individuals stay close to the bottom and often will not rise to the surface once shocked. This effect would be compounded in swiftly flowing streams, where electro-shocked fish are quickly swept into rocky interstitial spaces. As a consequence, snorkeling was elected over electro-fishing for survey in the current study. The Wet Tropics streams investigated were well suited for snorkel surveys in that they were clear and shallow, with fish easily seen in the water column and on the benthos. In addition, a previous pilot study indicated the reliability of single-pass snorkelling to estimate abundances of amphidromous species such as sicydiine gobies [25]. Fish presence was recorded by a single-pass snorkeling survey of each stream in a continuous fashion [25]. The majority of species were active during the day and were easily detected using this method. Cryptic and nocturnal species such as eels and eleotrids were detected by investigating rocky interstitial spaces with the aid of underwater torches. Snorkel surveys commenced at the end of tidal influence and continued upstream, finishing at elevations of 320 m above sea-level (a.s.l.), 420 m a.s.l, 60 m a.s.l., 110 m a.s.l. and 120 m a.s.l. in Noah Creek, Pauls Pocket Creek, Un-named Creek 1, Un-named Creek 2 and Un-named Creek 3, respectively. Surveys of Pauls Pocket Creek and Un-named Creek 3 continued to their source. Surveys of Noah Creek and Un-named Creek 2 did not continue past the indicated elevations due to the difficulty of the terrain, while the survey of Un-named Creek 1 was cut short due to inclement weather. Both Noah Creek and Pauls Pocket Creek had a small estuarine component, which was not surveyed thoroughly using this method due to the potential presence of estuarine crocodiles. Surveys were conducted between December 2009 and May 2010. Streams were surveyed by three researchers side-by-side with each snorkeler viewing 3−5 m of stream width. Where braiding of the stream occurred, researchers were allocated to stream braids. If more than three braids occurred in parallel, researchers snorkeled multiple braids so that all braids were surveyed. Where pools were greater than 15 m in width, two researchers surveyed the 5 m adjacent to each bank whilst the third researcher zig-zagged through the wider mid-stream section [25]. At the end of each riffle-run or pool section a record was made of each species observed. Most species were identified visually, checked against Allen *et al*. [30] and when an uncommon or a new species record for Australian was encountered, specimens were collected by dip-net, euthanised using 80 mg.L^−1^ of Aqui-S (Aqui-S NZ Ltd, Lower Hutt, New Zealand) and stored in 70% ethanol. These preserved specimens were identified by the Indo-Pacific IUCN freshwater fish taxonomists Dr Helen K. Larson and Dr Philippe Keith.

### Data analyses

In order to test the hypothesis that the fish community of coastal Wet Tropics streams were more similar to those of island streams than adjacent higher order continental rivers and their stream networks, the following analyses were undertaken.

**Figure 2:**
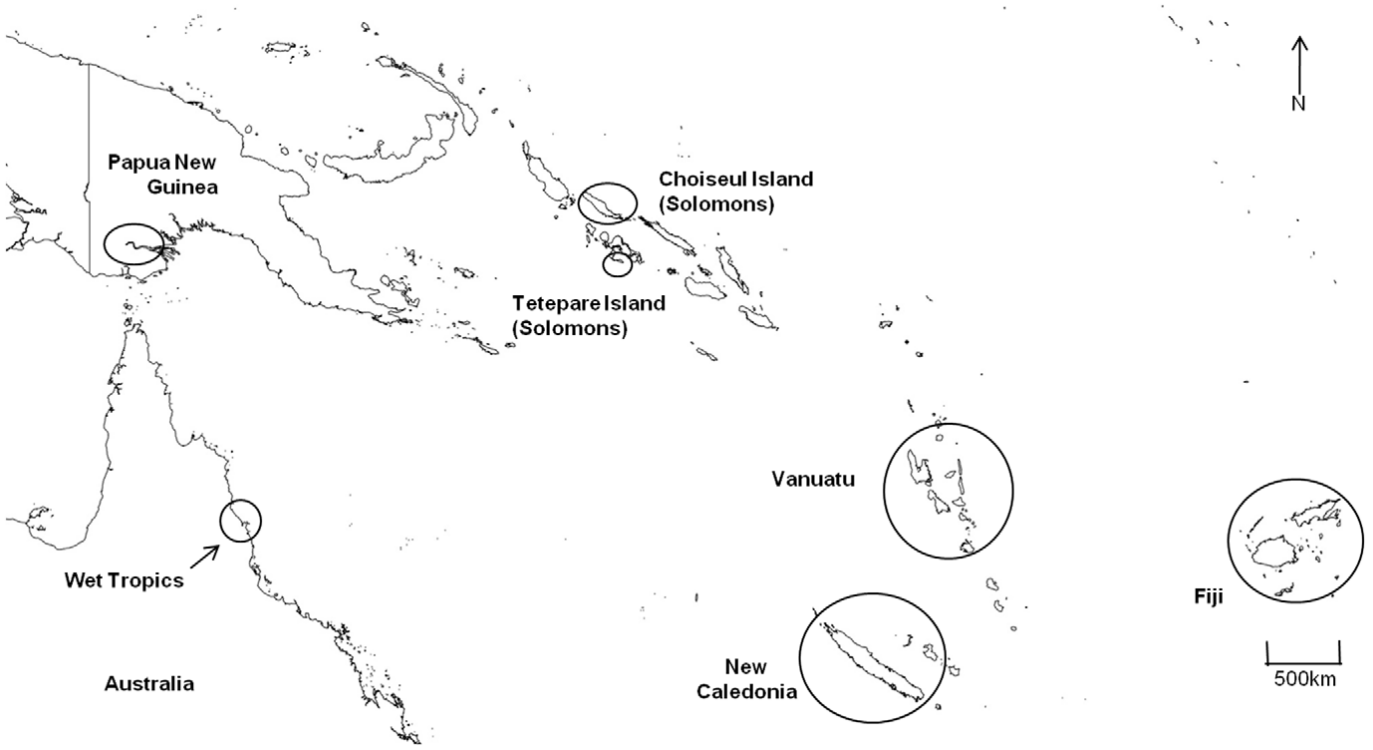
Map of the West Pacific freshwater fish communities used in analyses. Map indicating the Wet Tropics of north-eastern Australia, in relation to Papua New Guinea and West Pacific island groups. Open circles denote regions were freshwater fish communities were included for analyses.

First, the fish community data collected from 1) coastal Wet Tropics streams (CWTS) were compared with detailed presence/absence lists from 2) West Pacific island streams (WPIS) (Fig. 2) [2], [31], [32], [33], [34] (data also supplemented from www.fishbase.org), 3) Wet Tropics rivers (stream order ≥5) (WTR) [35] and 4) Papua New Guinea Rivers (PNGR) [36], [37]. It could be argued that a large dissimilarity in community structure was expected in the latter two comparisons, due to the dichotomy in physical size between the study streams and these larger continental rivers. Therefore, to control for these size differences, the fish community of coastal Wet Tropics streams was also compared to 5) Wet Tropics streams which drain into large rivers (WTS) [38], [39]. These adventitious coastal streams were of a similar magnitude in terms of physical size (stream order ≤3), steep gradient, substrate, flow and intactness to CWTS, but differ in that they flowed into larger rivers (stream order ≥5) with a significant estuarine zone. Unfortunately, an equivalent comparison could not be replicated for streams of Papua New Guinea rivers, as only species lists of entire catchments could be sourced from the literature. Only species which were known to have an obligate freshwater component to their life history were included in the analysis.

Second, a similarity matrix using the Bray-Curtis similarity coefficient [40] was generated and sites were graphically displayed in a dendrogram using agglomerative cluster analysis with group-average linking. These data were also ordinated using multidimensional scaling (MDS). Third, sites were pooled into *a priori* groups as detailed above and subjected to a non-parametric, one-way analysis of similarity (ANOSIM), using permutations (999) to assess average rank similarity between *a priori* groups. ANOSIM generates an *R* value, which is a measure of the similarity between groups. An *R* value of 1 indicated that a member within an *a priori* selected group was more similar to other members of that group than to members of any other group. All analyses were undertaken using the software package PRIMER [41].

### Prevalence of amphidromous and non-diadromous freshwater fish species

The *a priori* groups outlined above were also assessed individually to determine the proportion of the freshwater fish community that was a) non-diadromous and b) displayed an amphidromous life history. On the rare occasion that a species could not clearly be assigned to one of the categories, a judgement was made based on the life history of its congeners.

## Results

### Species composition of coastal Wet Tropics streams

Of the 26 species recorded during this study (Table 2) and a smaller pilot study [25], ten species (Anguilla marmorata, Dorichthys sp., Awaous ocellaris, Sicyopus discordipinnis, Smilosicyopus sp., Stiphodon atratus, Stiphodon birdsong, Stiphodon rutilaureus, Stiphodon semoni and Stenogobius cf genivittatus) represent first records for Australia. Three of these species are undescribed (Dorichthys sp., Smilosicyopus sp. and Stenogobius cf genivittatus) with a further three recorded species (Gymnothorax polyuranodon, Sicyopterus lagocephalus and Kuhlia marginata) being rarely encountered in Australia previously.

**Table 2:** Species richness of coastal north-east Australian Wet Tropics streams.

Family	Species	Status*	Noah Creek∧	Pauls Pocket Creek∧	Un-named Creek 1∧	Un-named Creek 2∧	Un-named Creek 3∧
Anguillidae	*Anquilla marmorata*	B#	1	1	1	0	0
Anguillidae	*Anguilla reinhardtii*	A#	1	1	1	1	1
Muraenidae	*Gymnothorax polyuranodon*	A#	1	0	0	0	0
Syngnathidae	*Hippichthys* sp.	A	1	0	0	0	0
Syngnathidae	*Doryichthys* sp.	B	0	1	0	0	0
Ambassidae	*Ambassis miops*	A#	1	1	1	0	0
Terapontidae	*Mesopristes argenteus*	A#	1	0	1	0	0
Kuhliidae	*Kuhlia marginata*	A#	1	1	1	1	0
Kuhliidae	*Kuhlia rupestris*	A#	1	1	1	1	1
Lutjanidae	*Lutjanus argentimaculatus*	A#	1	0	1	0	0
Gobiidae	*Awaous acritosus*	A#	1	1	1	1	0
Gobiidae	*Awaous ocellaris*	B#	0	1	1	1	0
Gobiidae	*Glossogobius* sp. 1	A#	1	1	1	1	0
Gobiidae	*Schismatogobius* sp.	A#	0	1	0	0	0
Gobiidae	*Sicyopus discordipinnis*	B#	1	1	0	0	0
Gobiidae	*Smilosicyopus* sp.	B	0	1	0	0	0
Gobiidae	*Stiphodon atratus*	B#	1	1	1	0	0
Gobiidae	*Stiphodon semoni*	B#	1	1	0	1	1
Gobiidae	*Stiphodon rutilaureus*	B#	1	1	1	1	0
Gobiidae	*Stiphodon birdsong*	A#	0	1	0	1	0
Gobiidae	*Stenogobius* cf *genivittatus*	B	0	0	1	0	0
Gobiidae	*Redigobius chrysosoma*	A#	1	0	0	0	0
Gobiidae	*Redigobius bikolanus*	A#	0	1	0	1	0
Eleotridae	*Eleotris fusca*	A#	1	1	1	1	0
Eleotridae	*Mogurnda adspersa*	A	1	0	0	0	0
Eleotridae	*Hypseleotris compressa*	A#	1	1	1	0	0
-	**Total**	**-**	**20**	**20**	**15**	**12**	**4**

*A - previously recorded in Australia; B - new species occurrence to Australia, i.e. from Thuesen [23], [24], Ebner & Thuesen [25] and current study. # Species shared with Pacific island groups.

∧0 = species absent from site; 1 =  species present at site.

**Figure 3:**
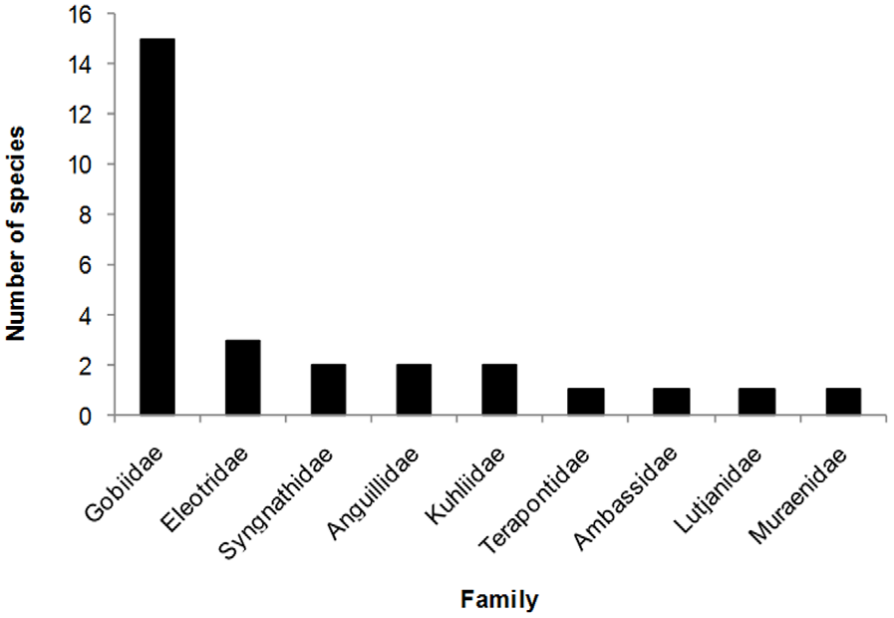
Species richness of family groups in coastal north-east Australian Wet Tropics streams.

The two larger streams, Pauls Pocket Creek and Noah Creek, were the most speciose systems (20 species each) while Un-named Creek 1 and Un-named Creek 2 contained 17 and 12 species, respectively. Species richness was much lower in Un-named Creek 3 (4 species), the smallest system investigated with a catchment area of 0.4 km^2^ (Table 1). Nine families were recorded with the Gobiidae being the most speciose (Fig. 3). This family contributed 54% of the fish fauna (15 species), of which almost half the species belonged to the subfamily Sicydiinae (7 species). Within this subfamily, the genus *Stiphodon* contained four species (Table 2). Species recorded only from Noah Creek were *Gymnothorax polyuranodon, Mesopristes argenteus*, *Lutjanus argentimaculatus*, *Mogurnda adspersa* and *Redigobius chrysosoma*. Species unique to Pauls Pocket Creek were *Smilosicyopus* sp., *Schismatogobius* sp., and *Doricthys* sp. Un-named Creek 1 had one unique species, *Stenogobius* cf *genivittatus*. Un-named Creek 2 and Un-named Creek 3 had no records of unique species.

### Sub-regionalisation of West Pacific streams

**Figure 4:**
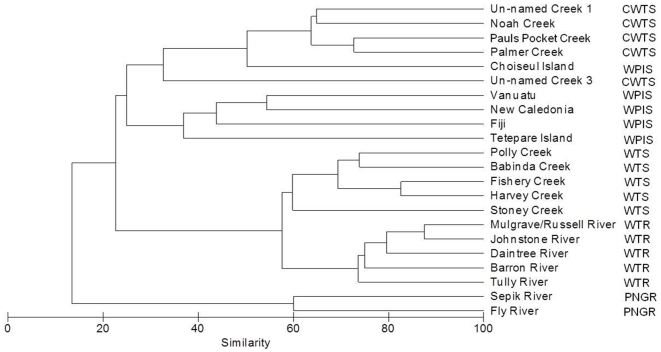
Phenogram of West Pacific freshwater fish communities. Phenogram derived from a Bray-Curtis similarity matrix of presence/absence data of freshwater fish fauna from sub-regions of the West Pacific (Key: **PNGR**  =  Papua New Guinea rivers, **WPIS**  =  West Pacific island streams, **WTR  = ** Wet Tropics rivers, **WTS**  =  Wet Tropics streams, **CWTS**  =  coastal Wet Tropics streams).

A cluster analysis of the Bray-Curtis similarity matrix data from all sites indicated the grouping of three major clusters: 1) PNGR 2) WTR and WTS 3) CWTS and WPIS (Fig. 4). Within cluster 2), WTR and WTS formed two separate sub-clusters, respectively. Sites within cluster 3) did not conform strictly to *a priori* designations.

**Figure 5:**
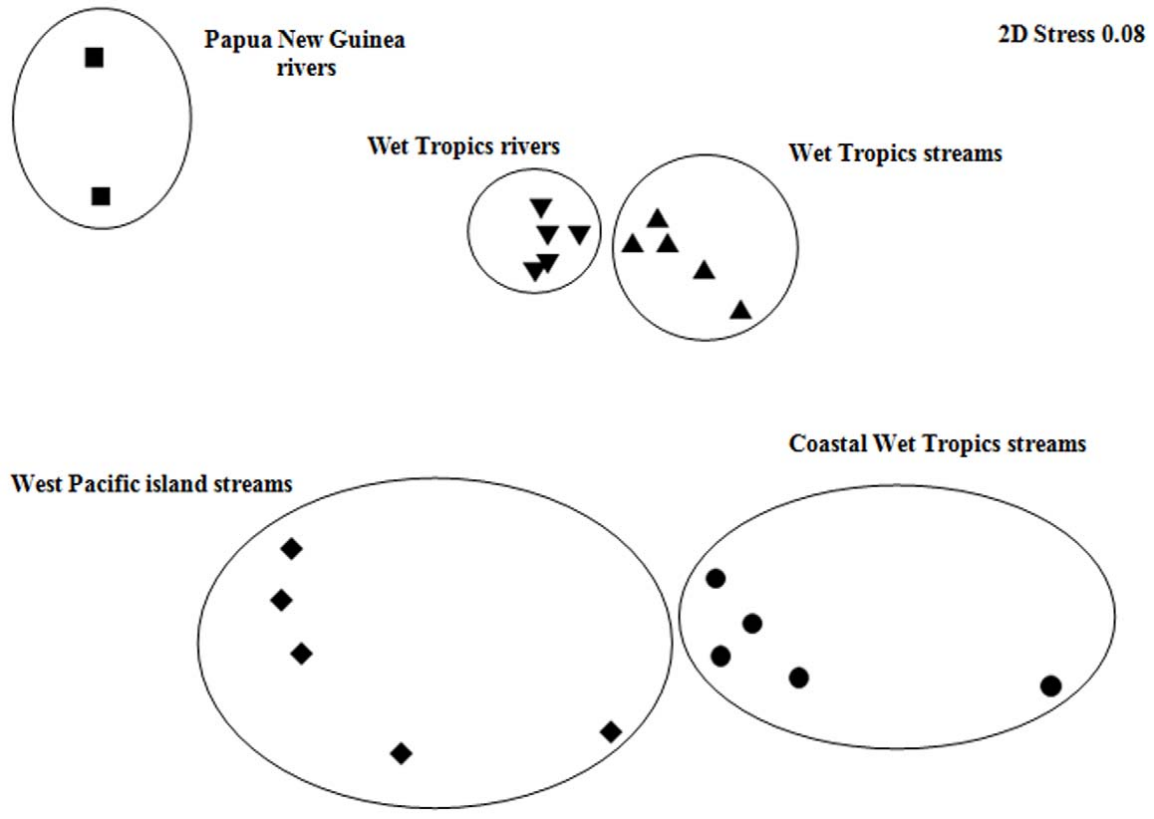
MDS ordination of West Pacific freshwater fish communities. Two-dimensional MDS ordination of freshwater fish assemblage presence/absence data for sub-regions of the West Pacific.

An MDS ordination of sites suggested that the fish fauna of CWTS grouped closely together in the lower half of the plot, with the exception of the fauna from one site (Un-named Creek 3), which differentiated to the lower far right (Fig. 5). Only four species were present in the latter site (Table 2). Dispersed broadly to the left lower-middle of the ordination was WPIS, with Choiseul Island (Solomon Islands region) grouping closer to CWTS than sites from any other group (Fig. 5). Sites from WTR and WTS formed a distinct group at the mid-upper range of the plot). The fish fauna of PNGR was clearly differentiated to all other sites and occupied the upper left of the ordination.

ANOSIM revealed strong differences between *a priori* groups (global R = 0.76, p<0.05) (Table 3), with all pair-wise comparisons of *a priori* designations being significantly different (P <0.05). However, the fish fauna of CWTS is more similar to those of WPIS (R = 0.76), than to those of WTR (R = 0.98) and WTS (R = 0.89).

**Table 3:** ANOSIM of West Pacific fish communities.

Sub-region	PNGR	WPIS	WTS	WTR
WPIS	1*	-	-	-
WTS	1*	0. 868 **	-	-
WTR	1*	0. 996 **	0.818**	-
CWTS	1*	0.764 **	0.878**	0. 976 **

R-stat values for pairwise ANOSIM comparisons of the fish assemblages from differing sub-regions of the West Pacific. N.B. Significant differences are represented by *P>0.05, ** *P>*0.01. Global R = 0.852 *. (Key: **PNGR**  =  Papua New Guinea rivers, **WPIS**  =  West Pacific island streams, **WTR  = ** Wet Tropics rivers, **WTS**  =  Wet Tropics streams, **CWTS**  =  coastal Wet Tropics streams.

### Composition of amphidromous and non-diadromous freshwater fish species

**Figure 6:**
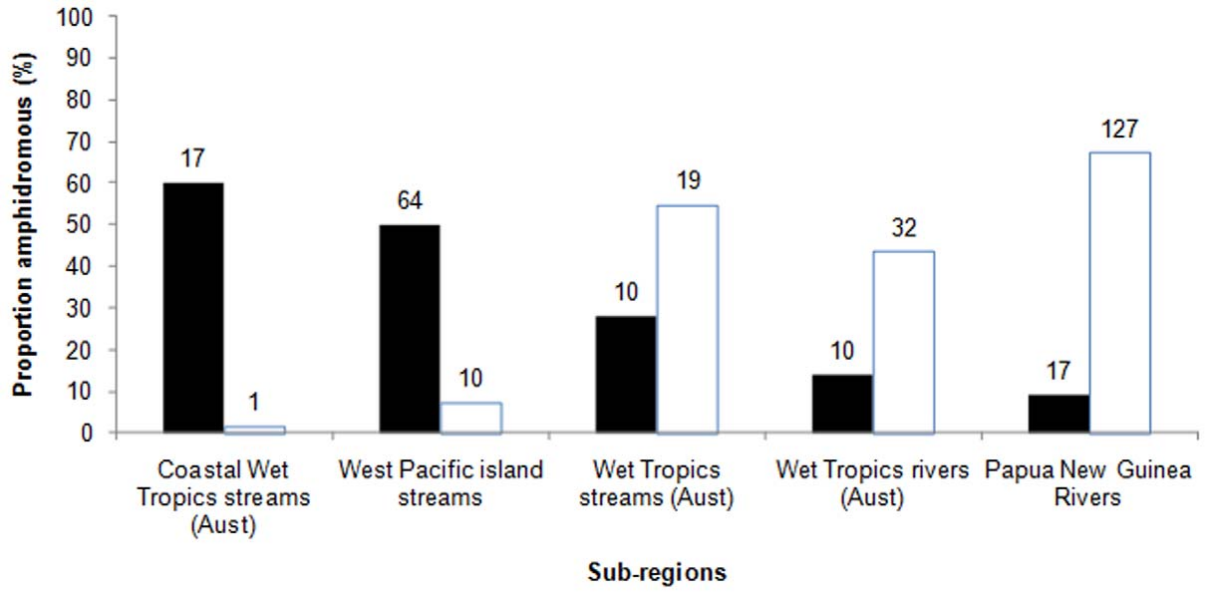
Life histories of West Pacific freshwater fish communities. Proportion of the freshwater fish community from differing sub-regions of the West Pacific that display an amphidromous life history (solid bars) and are non-diadromous (open bars).

Only one non-diadromous freshwater species, *Mogurnda adspersa*, was encountered during the survey. All other species were diadromous, with the sub-form amphidromy being the most prevalent life history strategy in CWTS, comprising 60% of the total fauna sampled (Fig. 6). This is high when compared to the proportion of amphidromous species found on adjacent WTS (28%) and WTR (15%), or PNGR (9%). Conversely, such a high level of amphidromy is comparable with WPIS regions (50%). The majority of species observed in CWTS have distributions that extend to Indo-Pacific islands (79%).

## Discussion

### Biogeography of coastal Wet Tropics streams

A major finding of the current study is that coastal Wet Tropics streams are faunally more similar to distant West Pacific islands (79% of species shared) than to nearby continental faunas of Wet Tropics rivers (60%). This can be attributed to two factors. First, coastal Wet Tropics streams are missing many of the non-diadromous freshwater fish which are common in the major river catchments of the Wet Tropics. These small streams are likely to be ephemeral in their hydrology over geologic timescales and vulnerable to dewatering from climatic fluctuations as is the case for streams of high Indo-Pacific islands [12], [13], [14]. As a consequence, non-diadromous species are unlikely to persist in these systems as, in contrast to amphidromous species, they have no marine larval phase for re-colonisation following local extirpation [12]. Second, the amphidromous species found in coastal Wet Tropics streams such as sicydiine gobies remain largely absent from major rivers of the Wet Tropics region due to the habitat requirements of their larvae. After hatching, free embryos of amphidromous gobiids require rapid transport to marine habitats [1], [4]. Experimental evidence suggests newly hatched larvae are better adapted physiologically to life in seawater, while prolonged exposure to freshwater retards development and increases mortality rates [1], [5], [6]. For example, *Sicyopterus lagocephalus* larvae die after 7 days if they have not reached the sea [42]. In addition, the larvae of amphidromous gobies feed on oceanic plankton [43]. Iguchi and Mizuno [4] suggested that early starvation of larvae limits reproductive success of fish located far from the sea, resulting strong selection for reproduction in steep, short and swiftly flowing coastal streams that drain directly into the ocean. Slower moving rivers with large lowland and estuarine components represent impassable barriers to migration, resulting in larval mortality [7].

Due to long geographical isolation and lack of extensive surface drainages, Australia's freshwater fish fauna is considered to be species-poor compared to other continents [21], [44]. The current study, along with a small pilot study [25] has added significantly to the number of freshwater fish recorded in Australia, and specifically in the north-eastern Wet Tropics (increased 15% from 56 species to 67 species). Of the 67 native freshwater fish species (i.e. obligate at some stage of their life history to freshwater) recorded for the Wet Tropics region of Australia, 51% are non-diadromous, 31% are amphidromous and the remainder exhibit other forms of diadromy. Fish faunas of the Indo-Pacific islands are characterised by a high proportion of amphidromous species, especially sicydiine gobies, which often comprise the highest levels of endemism and species richness [1]. Similarly, 61% of the species reported in this study have an amphidromous life history, with four sicydiine genera represented (*Sicyopterus, Sicyopus, Smilosicyopus* and *Stiphodon*). These genera contain seven species, one of which is undescribed (*Smilosicyopus* sp.).

It is important to note that the current study compares datasets which were collected using a variety of survey methods such as electrofishing, seine netting and snorkel. For instance, coastal Wet Tropics streams were snorkel-surveyed (this study) in a continuous fashion along the stream-continuum. In contrast, surveys of Wet Tropics rivers and their stream networks have mostly employed electrofishing to sample fish communities at a river catchment scale [18], [22]. It may be argued that this dichotomy in sampling regime significantly influences the pattern of biogeography described. For example, the patchy intra-stream distribution of some amphidromous species (e.g. *Stiphodon* spp. [25]) may not be intercepted by coarse-resolution survey designs. This may be true, however, we suggest the findings presented here are generally representative of the true fish community for a number of reasons. First, a major result, underpinning the faunal dissimilarity between coastal Wet Tropics streams and Wet Tropics rivers, is the conspicuous absence in the former of common non-diadromous species of the region (such as those belonging to the families Plotosidae, Atherinidae, Melanotaenidae, Pseudomugilidae and Terapontidae). If these species were present, our intensive survey design would easily have detected their occurrence. Second, coarse-scale electrofishing surveys are commonly used in Pacific Islands to collect amphidromous species such as sicydiine gobies [32], [33], [45] and it is reasonable to suggest that if these species were common in Wet Tropics rivers, previous surveys would have detected them. Third, the large estuarine components of Wet Tropics rivers may exclude many amphidromous species, whose larvae need immediate access to the sea.

### Biogeography of sicydiine gobies in the Pacific

McDowall [46] states that “amphidromous species seem to do poorly on continental lands, in the sense that continents seldom have them, especially lacking the sicydiine gobies”. Exceptions to this pattern are species of sicydiines found in continental South America, eastern Asia, India and Africa [47], [48], [49], [50]. The discovery of four sicydiine genera on mainland Australia does not alter the fact that sicydiine gobies are mostly found on island archipelagos but highlights their ability to colonise areas of suitable habitat, regardless of whether these streams occur on a continental or island landmass. The more critical factor in delimiting their biogeography is their inability to colonise river systems with large estuarine components due to larval starvation. As a consequence, they are rarely encountered on continents where steep, coastal stream systems are lacking.

The Pacific is known to be the centre of species diversity for sicydiines and may be the centre of origin for this subfamily [8], [51]. Patterns of sicydiine biogeography are likely to be influenced by a number of important factors including pelagic larval duration [52], and the direction of oceanic currents, which can constrict populations residing in ‘downstream’ extremities to sinks [12]. How Australian sicydiine populations fit into the meta-population structure of the wider Pacific community is unknown. However, it is likely that Australia represents a ‘downstream’ sink for populations of sicydiines, in comparison to diverse island groups of high endemism, due to the following lines of evidence. First, the numbers of individuals observed in the current study (Ebner and Thuesen, unpublished data) and the previous pilot study [25] are very small, with surveys detecting only a few individuals of some species. In contrast, on some Pacific islands, schools of migrating juvenile sicydiines can be so great that they form artisanal fisheries for indigenous inhabitants [53]. Second, none of the sicydiines recorded in Australia are endemic, with preliminary phylogenetic analyses indicating all species (including the undescribed *Smilosicyopus*) are a subset of the closest island archipelago regions of Papua New Guinea and the Solomon Islands (P. Keith, unpublished data). Lastly, the lack of endemic sicydiines encountered in the current study is unsurprising, as it is likely Australian populations have undergone repeated and complete extirpation during historical periods of lowered sea-levels. In contrast to most island streams that drain into deep oceanic water, coastal Wet Tropics streams drain onto the wide and shallow Great Barrier Lagoon (∼30 m below sea level). During the Pleistocene, glacial-interglacial cycles have resulted in sea levels fluctuations up to 150 m below current levels [54], resulting in the formation of large floodplains (>30 km wide) at the foot of these (ex) coastal ranges [55]. Amphidromous species (e.g. sicydiines), requiring steep gradient streams with immediate access to marine habitat, would fail to recruit due to larval starvation [35], [36] associated with the loss of stream-ocean connectivity. Presumably, during glacial minima these species can recolonise Australian streams by marine larvae sourced from Pacific high islands. In the Caribbean, fluctuating sea-levels and climatic shifts are likely to have resulted in the repeated extinction and recolonisation of amphidromous fauna [15], [56].

### Threats and conservation

Arguably the single most important factor in ensuring the conservation of amphidromous communities is the maintenance of stream-ocean pathways [1], [57]. Protecting natural patterns of flow is crucial for allowing trophic and/or gametic migrations between upstream and downstream reaches [58], [59], [60], [61]. Consistent base-flows facilitate rapid downstream migrations of newly hatched larvae which may otherwise starve in freshwaters [1], [4]. Consequently, amphidromous communities may be indirectly threatened by the construction of water abstraction infrastructure, which disrupt these flow regimes [62]. Undoubtedly the newly discovered sicydiine fish assemblage at Russell Heads (Un-named Creek 2) has not been factored into local water resource planning for the region. This should be redressed, to ensure water abstraction for urban use is managed in a manner that maintains minimal flows necessary for migratory amphidromous species. In addition to natural flows, the preservation of a fully functioning riparian zone is necessary to maintain the cooler water temperatures and high oxygen levels to which species are adapted [1]. Intact riparian vegetation also buffers against siltation of streams [63], which inhibits the growth of benthic algae that many sicydiine gobies graze on and prevents spawning through a lack of clean substratum for egg adhesion [33].

When small estuaries are present at the base of these small streams, their natural state should be conserved as they become important areas where species transit (i.e. larvae of amphidromous species exit to the sea, and post-larvae/juveniles enter to metamorphose and colonise streams) [1]. Introduced species such as tilapia (*Oreochromis mossambicus*) have been implicated in the decline of amphidromous species (including one of those species collected in the current study - *Stiphodon rutilaureus*) in Fijian streams [33]. Tilapia (*O. mossambicus* and *Tilapia mariae*) have been present in Australia since the 1970s and control options are limited [64]. While they do not currently occur in these study sites, they are present in adjacent larger rivers [18].

Lastly, aquarium collectors pose a serious and immediate direct threat to sicydiine gobies within Australia [25]. These species are vulnerable to over-harvesting for the aquaria trade as a result of their colourful appearance, interesting behaviours [65], [66], [67] and are easily collected by dipnet [25]. Given the small population sizes of these species discovered to date in Australian streams [25] and the close proximity of these populations to the regional centre of Cairns, we recommend that in addition to *Stiphodon* protective status be urgently reviewed for all sicydiine gobies in Australia.

### Conclusion

The current study has confirmed the presence of a mostly amphidromous fish community from north-eastern Australia. Coastal Wet Tropics streams are faunally more similar to streams of high islands located thousands of kilometres across the Pacific, than to neighbouring large rivers which exit into the sea as little as a few kilometres away. This can be attributed to 1) the ephemeral nature of these coastal Wet Tropics streams, which exclude many non-diadromous freshwater fish that are common in large rivers of the Wet Tropics 2) the habitat requirements of amphidromous larvae found in coastal Wet Tropics streams and Indo-Pacific islands, which are not met in large, slower flowing continental river systems. In the short term, a major conservation priority is the protection of sicydiine gobies from potential overharvest in Australia. In the longer term, assessment of the conservation value of this fauna will require knowledge of the distribution, abundance, taxonomy and population genetic structure (especially potential source-sink dynamics) of these species, to determine how this newly discovered continental fauna relates to that of streams elsewhere in the Indo-Pacific.
